# A study of the prevalence of diarrhoeagenic *Escherichia coli* in children from Gwagwalada, Federal Capital Territory, Nigeria

**DOI:** 10.11604/pamj.2014.17.146.3369

**Published:** 2014-02-28

**Authors:** Adebola Onanuga, Oluwatoyin Igbeneghu, Adebayo Lamikanra

**Affiliations:** 1 Department of Pharmaceutical Microbiology & Biotechnology, Faculty of Pharmacy, Niger Delta University, Wilberforce Island Bayelsa State, Nigeria; 2 Department of Pharmaceutics, Faculty of Pharmacy, Obafemi Awolowo University, Ile – Ife, Nigeria

**Keywords:** Prevalence, diarrhoeagenic Escherichia coli, diarrhoea, healthy, children

## Abstract

**Introduction:**

Diarrhoeagenic *Escherichia coli* (DEC) are major causes of diarrhoea in Nigeria. This study was conducted to determine the prevalence of diarrhoea caused by DEC within the Federal Capital Territory, Abuja, Nigeria.

**Methods:**

A total of 730 rectal swabs obtained from 201 children with diarrhoea and 529 healthy controls aged 0-24 months were cultured for the isolation of *Escherichia coli*. All *E. coli* isolates were investigated by PCR to determine their pathotype.

**Results:**

A total of 61 DEC strains were recovered at a rate of 18.4% and 2.6% from children with diarrhoea and healthy controls respectively. The DEC strains recovered were Enteroaggregative *Escherichia coli* (34.4%), Shiga-toxin producing Escherichia coli (31.1%), Enterotoxigenic *Escherichia coli*(18.0%), typical enteropathogenic *Escherichia coli* (15.0%) and Enteroinvasive *Escherichia coli* (1.6%). Shiga-toxin producing *Escherichia coli* andEnteroinvasive *Escherichia coli* were recovered only from children suffering from diarrhoea and the overall prevalence of DEC strains was significantly higher among the children with diarrhoea (P < 0.0001). The number of DEC strains obtained during the dry season was significantly higher (P = 0.012) than the number obtained in the rainy season.

**Conclusion:**

Diarrhoea caused by *E. coli* in the Nigerian children studied is associated with several diarrhoeagenic pathotypes and a significant proportion of the healthy children were found to harbour EAEC and ETEC strains. These asymptomatic carriers may be regarded as potential transmitters of infection to vulnerable children in the study area.

## Introduction

Diarrhoeal diseases are a global public health problem causing considerable morbidity and mortality among infants and children especially in the developing countries [1–2]. Diarrhoea is responsible for annual global deaths of about 2.6 million people mostly among African children under the age of 5 years [3] and the significant reduction in child mortality observed in recent years has been attributed to the practice of exclusive breast feeding as well as recourse to oral rehydration therapy in the treatment of diarrhoea [4].

Infections due to faecal-water contamination are caused by a host of enteric infectious agents including bacteria, viruses, fungi and parasites. Among the bacteria, at least six pathogenic of diarrhoeagenic *Escherichia coli* (DEC strains) are frequently detected worldwide. These pathotypes have been described based on the genes mediating the virulence factors associated with the diarrhoeal disease caused by them: Enteropathogenic *E. coli* (EPEC), Enterotoxigenic *E. coli*(ETEC), Shiga toxin-producing *E. coli* (STEC) or Enterohaemorrhagic *E. coli* (EHEC), Enteroaggregative E. coli (EAEC), Enteroinvasive E. coli (EIEC) and diffusely adherent E. coli (DAEC) [5–6].

The identification of DEC pathotypes is through the use of molecular methods for the detection of the genes responsible for mediating the substances responsible for pathogenicity. This is essential for identification and classification of DEC and is based on the presence of different chromosomal and/or plasmid-encoded virulence genes that are absent in commensal *E. coli*. Moreover, the prevalence and other epidemiological features of these pathogens as causative agents of diarrhoea vary from one region to the other, and even between and within countries in the same geographical area [7–8]. In Nigeria for example, only very few studies have investigated the microbiology of childhood diarrhoea and these have all been carried out in the South. This is a report of such a study carried out in the North-Central part of the country.

This study therefore investigated the prevalence of diarrhoeagenic *Escherichia coli* isolated from children in Gwagwalada, Federal Capital Territory, Nigeria as a means of determining the distribution of DEC associated diarrhoea in the North-Central part of Nigeria.

## Methods

### Study Population

A total of 730 children aged 0-24 months who reported to the paediatric unit of the University of Abuja Teaching Hospital, Gwagwalada with complaints of diarrhoea (with or without fever or other accompanying symptoms) and had not taken any antimicrobial agent in the preceding week were recruited into this study over a period of one year (1st April, 2008 to 31st March, 2009). The children in the same age range who reported for the immunization programme at the Township Clinic in the same town were regarded as apparently healthy children and used as controls in this study. Gwagwalada is a fast growing satellite town in the Federal Capital Territory, Abuja in the North Central Nigeria and the teaching hospital is a tertiary care hospital with a 500-bed capacity that offers a full range of services to people living mostly in the Middle Belt area of the country. The study was approved by the ethical committees of the participating institutions while the parents or guardians of the children gave informed consent and filled a questionnaire to provide demographic data and the breast-feeding pattern for each child.

### Specimen Collection

Faecal specimens were obtained as rectal swabs from each of the children in the study by inserting sterile cotton wool applicators to the rectums of the children. The faecal material thus obtained was transported to the laboratory in sterilized Cary Blair transport medium for the culture and isolation of *E. coli*. Rectal swabs were used in this study because they are more easily obtained from all the volunteers than the stool samples which are considered the gold standard for the identification of gastrointestinal (GI) tract colonization with bacteria. Moreover, Lautenbach et al. [9] have shown that rectal swabs are able to allow the cultivation of 90 - 100% faecal contaminants when compared to the faecal samples in the isolation of enteropathogens.

### Isolation and storage of E. coli

Each rectal swab from the subjects was inoculated onto the surface of MacConkey and Eosin Methylene Blue (EMB) agars (Oxoid, UK) within 24 hours of collection and streaked for isolated colonies. The organisms that produced characteristic discrete colonies after 24 hours of incubation at 37°C were streaked onto fresh sterilized Nutrient agar (Oxoid, UK) and identified by conventional biochemical IMViC reactions (Indole, Methyl red, Voges Proskauer, Citrate,Urease tests) [10]. The isolates that were positive to indole and methyl red tests but negative to voges proskauer, citrate and urease tests were identified as *E. coli*. All *E. coli* colonies from a single rectal culture with identical colony morphology, and biochemical properties were assumed to be identical while four to five E. coli isolates with different colony morphology that were positive to the conventional biochemical tests arising from a single rectal swab from each of the children were maintained in the laboratory in cryovials (Nalgene, USA) by cryopreservation at -70°C and also stored in nutrient agar slants at 4°C in a refrigerator for the investigation of the genes encoding pathogenicity at molecular level.

### Screening for Diarrhoeagenic E. coli genes

DNA from each confirmed E. coli isolate was extracted from the organism after a 24 hour incubation period on nutrient agar plates by suspending three colonies in 50 μl of deionized water and boiling for 10 minutes. This was cooled on ice for at least 2 minutes, followed by centrifugation at 3 RAM (3,000 per g for 5 minutes) using BIO-RAD Microcentrifuge (Model 16K) (BIO-RAD Laboratories, USA) to pelletise the cell debris. Exactly 2 μl of each test isolate's supernatant was used as the DNA template for Polymerase Chain Reaction (PCR) analysis. Two (2) μl of lysate from the reference strains EPEC E2348/69, EAEC O42, ETEC H10407, EIEC EDL1284, and STEC EDL931 which served as positive controls and *E. coli* K-12 DH5α which was the negative control and from all the *E. coli*-positive isolates were subjected to multiplex PCR with specific primers. This was with a view to detecting the following diarrhoeagenic virulence markers: eaeA(structural gene for intimin of EHEC and EPEC), *bfpA* (structural gene for the bundle-forming pilus of EPEC), stx1 and/or st×2 (verocytotoxin 1 and 2 of EHEC and STEC), *eltB* and/or *estA* (enterotoxins LT and ST of ETEC), *ipaH* (invasion-associated locus of the invasion plasmid found in EIEC and *Shigella*) and *pCVD* (the nucleotide sequence of the EcoRI-PstI DNA fragment of pCVD432 of EAEC) as described by Nguyen *et al*. and Aranda et al. [7, 11].

PCRs were performed using an optimized protocol which was carried out on ice with a 100 μl reaction mixture containing 10 mM Tris-HCl(Trisaminomethane hydrochloride) (10× Standard Reaction Buffer (pH 8.3) (New England Biolabs Inc. UK), 50 mM MgCl_2_ (New England Biolabs Inc. UK), 10 mM concentration of dNTPs (Madison, USA), 50 nmol each of PCR primers (Eurofins, USA), a 5 units per μl pure *Taq* DNA polymerase (Standard TaqMg-free Buffer) (New England Biolabs Inc. UK) and 2 μl of the DNA template. The optimal concentration of each primer pair in the reaction mixture was determined empirically. The PCR was carried out in a GeneAmp PCR system 9700 (Applied Biosystems, Singapore) with the following thermocyclingconditions: For PCR assay designated as “1”_ 50°C (2 min, 1 cycle); 95°C (5 min, 1 cycle); 40 cycles of 95°C (40 s), 58°C (1 min), and 72°C (2 min); and a final extension step at 72°C (7 min, 1 cycle); and for PCR assay designated as “2” (which was divided into PCR 3 and PCR 4) _ 50°C (2 min, 1 cycle); 95°C (5 min, 1 cycle); 40 cycles of 95°C (45 s), 50°C (1 min), and 72°C (1 min); and 72°C (7 min, 1 cycle) in a thermal cycler (GeneAmp PCR system 9700, AppliedBiosystems, Singapore). The PCR products (10 μl) were evaluated on a 1.5% (w/v) agarose gel (UltraPure Agarose; Invitrogen Life Technologies) at 100 mV for 60 minutes using BIO-RAD Power Pac 3000 (BIO-RAD Laboratories, USA) and a molecular weight marker (100 bp DNA Ladder; New EnglandBiolabs. UK) was run concurrently. The DNA bands were then visualized and photographed under UV light (using UVitec, UVisave; (Avebury, Cambridge UK) and Video copy Processor; Mitsubishi Electro, Malaysia) after staining the gel with ethidium bromide.

### Statistical Analysis

Frequencies and percentages were calculated for the study variables and the data obtained were compared with the use of a two-tailed Chi square and Fisher's exact tests. A P-value of less than or equal to 0.05 (P ≤ 0.05) was considered to be statistically significant.

## Results

A total of 730 rectal swabs from 201 children with diarrhoea and 529 apparently healthy of ages 0-24 months that were made up of 337 females and 393 males were examined for the presence of *E. coli* strains. Only 277 and 388 *E. coli* isolates recovered from the screened specimens from diarrhoea and control groups respectively were screened for possible virulent diarrhoeagenic genes. The analysis of the questionnaire on feeding pattern of all the children revealed that 76% of the children in age group 0-3 months were exclusively breast fed (Table 1).

**Table 1:** Analysis of the feeding pattern of children in the breast feeding age groups

Age Group	No. of children (%)					Total
	0 – 3 month	4 - 6 month	7 – 12 month	13 -18 month	19 – 24 month	
Feeding Pattern						
Exclusive Breast feeding	157 (75.9)	53 (41.1)	6 (3.1)	0	0	216
Breast milk + other foods	42 (20.3)	65 (50.4)	167 (85.2)	42 (36.2)	0	316
Formula milk + other foods	8 (3.9)	11 (8.5)	6 (3.1)	9 (7.8)	0	34
Other foods only	0	0	17 (8.7)	65 (56.0)	82 (100)	164
**Total**	207	129	196	116	82	730

The diarrhoeagenic *E. coli* strains were detected at an overall rate of 18.4% (n=51) and 2.6% (n=10) from the isolates recovered from diarrhoea and control groups respectively. They comprised of Enterotoxigenic *Escherichia coli* (n =11), Typical enteropathogenic Escherichia coli (n =9), Enteroaggregative *Escherichia coli* (n =21), Shiga-toxin producing Escherichia coli (n =19) and Enteroinvasive *Escherichia coli* (n =1) (Table 2). The overall prevalence of DEC strains was significantly higher among the isolates from the children with diarrhoea (P < 0.0001).

**Table 2:** Frequency of DEC among the diarrhoea group and other children's categories

Diarrhoeagenic *E. coli* (DEC)	Pathotype Genes	Subjects’ Categories		P-value
		Diarrhoea N = 277	Controls (Non-Diarrhoeal) N = 388	
ETEC		7	4	0.2156
LT-ETEC	*LT*	4	3	0.4584
ST-ETEC	*ST*	3	0	0.0399*
LT & ST	*LT &ST*	0	1	1.0
EPEC (Typical)	*bfpA, eae*	8	1	0.0049*
EIEC	*IpaH*	1	0	0.4165
STEC	*Stx*_*1*_ *&Stx*_*2*_	19	0	<0.0001*
EAEC	*EAEC*	16	5	0.0014*
**Total**		51	10	<0.0001*

* Statistically significant (*P* ≤ 0.05).

The highest proportion of DEC (15.4%) was detected among the children in age group 4-6 months whilst those in age groups 0-3 months and 22-24 months provided the lowest number of DEC isolates. The EPEC strains were found only among the children in age group 0-9 months whilst ETEC strains were most frequently encountered among the children aged 16-18 months. A total of 87.5% of EAEC and 78.9% of STEC strains were significantly found among children older than 3 months (P < 0.00001) in the children with diarrhoea.

The detection of DEC in the study year was found to be highest in March (27.1%), moderate in May (14.5%) and December (15.0%) but lowest in June with no DEC strain recovered. The observed difference in the monthly prevalence of the DEC was highly significant in the month of March (P = 0.00002) (Table 3). The ETEC and EPEC strains were significantly detected in the dry season (P = 0.003 and 0.015, respectively) and the overall prevalence of diarrhoeagenic *E. coli*was significantly higher during the dry season (November-March) than the rainy season (April-October) (P = 0.012) (Table 4).

**Table 3:** The monthly trend of diarrhoeagenic *E. coli* (DEC)

Month	No. of Isolate Screened	Diarrhoeagenic E. coli (DEC) (%)	P-value
January	49	4 (8.2)	1.0
February	34	3 (8.8)	1.0
March	59	16 (27.1)	0.00002*
April	92	6 (6.5)	0.4377
May	55	8 (14.5)	0.2177
June	16	0	0.3849
July	52	1 (1.9)	0.0753
August	97	7 (7.2)	0.5706
September	47	3 (6.4)	0.6097
October	59	4 (6.8)	0.6404
November	85	6 (7.1)	0.5518
December	20	3 (15.0)	0.4169
**Total**	665	61	

* Statistically significant (*P* ≤ 0.05).

**Table 4:** Seasonal variation of the diarrhoeagenic *E. coli* pathotypes

DEC pathotype	Season (%)		P-value
	Dry N = 247	Rain N = 418	
ETEC	9 (3.6)	2 (0.5)	0.003*
EPEC	7 (2.8)	2 (0.5)	0.015*
EIEC	0	1 (0.2)	1.0
STEC	11 (4.4)	8 (1.9)	0.089
EAEC	5 (2.0)	16 (3.8)	0.254
**Total DEC**	32 (13.0)	29 (6.9)	0.012*

* Statistically significant (*P* ≤ 0.05).

## Discussion

Diarrhoeagenic *Escherichia coli* strains are pathogens of public health importance affecting both adults and children worldwide. Most diarrhoeal cases in children under 5 years of age have been shown to be due to diarrhoeagenic *E. coli* in which ETEC, EAEC and EPEC strains are the most prevalent in the developing countries [8, 12].

In this study, the frequency of recovery of DEC strains was significantly higher among the children with diarrhoea (18.4%) than those without diarrhoea (2.6%). This significant association of DEC with diarrhoea in the study environment is as it has been reported by other previous studies carried out in other locations such as Ghana [13] and Brazil [14]. Similar results have also been reported from the south eastern part of Nigeria [15]. This study however recorded a lower rate of recovery of DEC from the diarrhoeal children than these other studies where the rate of recovery ranged between 21.4% in India [16] and 36.8% in Brazil [14]. The low prevalence of DEC among the diarrhoeal cases also suggests that other causative agents (such as rotavirus, protozoan *Giardia, Salmonella* and *Shigella species*) that were not investigated in this study might be other causes of the diarrhoea [17, 18]. The recovery of DEC from only 2.6% of the apparently healthy subjects in this study environment suggests that these pathogenic organisms are rarely encountered in healthy children and the few from which thediarrhoeagenic *E. coli* were isolated might be recovering from diarrhoea or were in the pre-symptomatic stage of the infection.

The distribution of DEC among the various children age groups in this study showed that children in age groups 0-3 and 22-24 months had the lowest prevalence of DEC infections. This observation suggests that the children were protected in the first three months of their life, when they were mostly being breast fed, by the antibodies in breast milk and later on by their own acquired immunity [19, 20]. The prevalence of DEC was therefore found to be highest among the children in age groups 4-9 and 16-21 months age groups in which immunity conferred by breast milk had been waned before their capacity to mount an effective immunological response is developed [21–22]. In addition, children within the age 16 to 21 months were found to have been weaned and their exposure to adult foods constitutes a risk factor since such foods are more likely to be contaminated by microorganisms than breast milk.

The results of this study showed a seasonal variation in the prevalence of DEC infection in the environment. It was observed that the overall prevalence of infection was significantly higher during the dry season than the rainy season (P = 0.012). The peak prevalence was observed in March, a period considered as one of the driest and hottest months in the study area, when there is a shortage of potable water in area in which municipally treated water is not available. In addition, the high temperatures which are characteristic of this period are favourable to the proliferation of infectious agents in the tropics [23]. This observation is similar to previous studies on seasonal variation of DEC infection reported by El Metwally et al. in Egypt [24] and Albert et al. in Kuwait [25]. The four common pathotypes of E. coli which include ETEC, EPEC, STEC and EAEC were detected in this study. Their frequencies of recovery were significantly higher in the diarrhoea group than in the non-diarrhoeal category. This supports the findings of other investigators in some other developing countries [7, 13, 16, 24].

Enteroaggregative *E. coli* (EAEC) strains were the most frequently recovered DEC pathotypes in this study. Their prevalence was found to be significantly higher among the children with diarrhoea than those without diarrhoea (P = 0.0014) and the children in the 0-3 month age group were the least affected (12.5%). EAEC was the pathotype with the highest prevalence (50.0%) among the children without diarrhoea and 80% of the children in these categories were from the 0-6 month age group. This observation shows EAEC as an increasingly recognized cause of diarrhoea illness among children in developing countries as reported by other workers [14, 16, 26]. The high rate of the isolation of EAEC in this group of subjects and the significantly higher prevalence (80.0%) among the 0-6 month age group without diarrhoea suggests that immunity to this pathotype is widely developed at an early age and supports the postulation of Okeke [27] that infection with EAEC before weaning was unlikely to lead to diarrhoea.

Shiga toxin-producing *Escherichia coli* (STEC) infection causes acute and bloody diarrhoea. The STEC strains recovered in this study were made up of 8 strains with St×1, 3 with St×2 and 8 with both St×1 and St×2 genes. They were only detected from the isolates from children with diarrhoea at a prevalence rate of 6.9% and were frequently found in children within the 6-24 month age group. This observed prevalence of STEC strains disagrees with the findings of previous workers who reported very low or no isolation in children with diarrhoea [15, 16, 24] while it is similar to reports of Alikhani et al. [28] in Iran and Garcia et al. [14] in Brazil who recovered STEC of 8.7% and 7.4% respectively from children with diarrhoea. The reason for the isolation of this pathotype within the study environment is not known and requires further investigation.

The ETEC strains in this study were made up of 7 strains bearing genes encoding LT, 3 strains with ST and 1 strain with both LT and ST (two enterotoxins). The ST strains were only found in the diarrhoea group while the only isolate that produced both LT and ST was from an apparently healthy child. This higher prevalence of LT-ETEC over ST-ETEC observed in the children of ages 0-24 months has been previously reported by Valentiner-Branth *et al*. [29] in Guinea and El Metwally *et al*. [24] in Egypt while the detection of ETEC strains producing the two enterotoxins (LT and ST) have been reported by Okeke [30] in Ile-Ife, Nigeria and El Metwally *et al*. [24] in Egypt. ETEC diarrhoea in this study was encountered mainly in children in the 4-18 month age group, whilst none of the children in the 0-3 month age group had ETEC diarrhoea but harboured the LT and ST strains. The high prevalence of ETEC diarrhoea among the older children may be due to the fact that children in these groups lose the immunity conferred on them through the antibodies passed to them from the breast milk of their mothers.

Enteropathogenic *E. coli* are currently classified into two sub-categories, that is, typical and atypical EPEC. While typical EPEC are established pathogens, thepathogenicity of atypical EPEC is still a subject of debate [31]. The EPEC strains detected in this study were all typical EPEC and they were found to be significantly higher among children with diarrhoea than those without diarrhoea. Of the 9 isolates of EPEC recovered, only one of them was found in a child without diarrhoea confirming the association between typical EPEC and diarrhoeal disease. The only EPEC strain in the non-diarrhoeal group was found in a 6 month old child whilst one of the strains in the diarrhoea group came from a 3 month old child. This shows the protective power of mother's partial immunity and exclusive breastfeeding on the children against EPEC diarrhoea [32]. All the EPEC strains detected in this study were from the children in the 0-9 month age group thus suggesting that EPEC is one of the main causes of infantile diarrhoea [16].

The isolation of 1(0.6%) EIEC isolate from diarrhoea group in this study agrees with previous studies in the detection of very low rate of this pathotype that is known to cause diarrhoea symptoms similar to shigellosis in adults and children [14, 24]. The lack of epidemiological attention to EIEC is related to the low incidence of this pathogen as a cause of diarrhoea when compared to other pathotypes of diarrhoeagenic *E. coli*. As pointed out by Vierra *et al*. [33], researchers have not reported the isolation of these organisms from patients with diarrhoea. One of the reasons why the isolation rate of this type of DEC is low is related to the fact that they are missed when only lactose fermentation is used as a preliminary screening tool for diarrhoeagenic *E. coli* since over 70% *E. coli* in this group do not ferment lactose [27].

The findings from this study corroborate the reports in literature on the protection conferred on infants by breast-milk against infectious diseases. Breast-milk is considered to be the best source of nutrients and immunological factors needed for infants to grow and resist infections in the early stages of life [34]. The practice of exclusive breastfeeding (EBF) in the first 6 months of life and continued breastfeeding up to the 11th month of birth has been identified as the single most effective preventive intervention in reducing child mortality due to diarrhoea and respiratory tract infections [35]. Hence the recommendation that EBF should be practiced by nursing mothers globally. The results of this study however showed that in the Federal Capital Territory of Nigeria, only 62.5% of infants in the age bracket 0-6 months were exclusively fed on breast-milk and only 17.4% of children above the age of 6 months were still being breast fed. The figure of 62.5% for exclusive breast feeding, in this study, whilst lower than the desired figured is however well in excess of 16.4% and 32.2% which have been reported from other parts of northern Nigeria [36, 37]. Other studies in Nigeria also show that compliance with exclusive breast feeding is significantly lower than desired [38, 39]. Given the effectiveness of exclusive breast feeding to the reduction of infant morbidity and mortality due to diarrhoea; it is important that efforts to impress desirability of breast feeding on mothers be intensified and compliance monitored throughout Nigeria and indeed other developing countries.

## Conclusion

The results of this study suggest that diarrhoeagenic *E. coli*, especially STEC, EAEC and EPEC are strongly associated with childhood diarrhoea within the study environment whilst both EAEC and ETEC strains were recovered from apparently healthy children who could then be sources of the transmission of these pathogens to other children. Such transmission could however be prevented through the widespread application of hygienic practices, the training for which should be carried out by appropriately trained public health workers.
